# Euphrasia Officinalis

**Published:** 1891-05-09

**Authors:** 


### THERAPEUTICS.

### EUPHRASIA OFFICINALIS.

The Euphrasia officinalis, or eyebright, is a small annual plant found all over Europe and the United States. It has no smell, but an astringent, somewhat bitter taste, although the tincture has a pleasant aromatic taste, and is well taken by children. Enz found it contained a small quantity of a volatile oil, and acrid and bitter substance, mannite and glucose, and a form of tannic acid.

It is a very old remedy; at one time, though long since discarded, a common popular remedy for certain eye affections, as shown by the names "eyebright," "augentrost," "casses-lunettes," it bears in different countries. We have used it chiefly as a local application in catarrhal conditions of the mucous membranes of the eye and nose in the form of a lotion made of the tincture diluted with water. We have found this lotion dropped in the eye produces remarkably good results in the slight forms of conjunctivitis in young children; on the other hand, in other cases its action was apparently *nil.* An adult male patient, who, suffering from subacute conjunctivitis, persevered with the euphrasia drops for a fortnight without the slightest relief, but was perfectly cured after the use of drops of sulphate of zinc, two grains to the ounce. The inefficacy of the remedy in this case, while exceptional, is sufficient to show that it is uncertain in its action. Dr. Garland has recently published four cases of coryza in which various remedies had been tried without producing any relief, but all of which were relieved with surprising promptitude by tincture of euphrasia in doses of ten drops internally. The cases were all seen in the earlier stages, and the remedy seemed to have been successful in aborting the attacks. He found that when administered at the onset of the attack it apparently entirely arrested the flow of mucus from the nasal mucous membrane; but that in the later stages, when the discharge had become muco-purulent, it did not affect the course of the affection. Dr. Garland has found it most efficient when given internally to children. It would appear from this that when given internally better results may be expected than when used as a local application.

